# Leaf rolling dynamics for atmospheric moisture harvesting in wheat plant as an adaptation to arid environments

**DOI:** 10.1007/s11356-022-18936-2

**Published:** 2022-02-25

**Authors:** Sabah Merrium, Zulfiqar Ali, Muhammad Hammad Nadeem Tahir, Muhammad Habib-ur-Rahman, Sadia Hakeem

**Affiliations:** 1 Institute of Plant Breeding and Biotechnology, MNS-University of Agriculture, Multan, 60000 Pakistan; 2 Institute of Crop Science and Resource Conservation (INRES), Crop Science Group, University of Bonn, Bonn, Germany; 3 Department of Agronomy, MNS-University of Agriculture, Multan, 60000 Pakistan

**Keywords:** Abaxial, Adaxial, Atmospheric water, Contact angle, Drought, Dynamics, Hydrophilic, Root zone, Wheat

## Abstract

Plant species surviving in the arid regions have developed novel leaf features to harvest atmospheric water. Before the collected water evaporates, it is absorbed and transported for storage within the tissues and move toward the root zone through the unique chemistry of leaf structures. Deep insights into such features reveal that similarities can be found in the wheat plant. Therefore, this study aimed to evaluate the leaf rolling dynamics among wheat genotypes and their relationships with moisture harvesting and its movement on the leaf surface. For this purpose, genotypes were characterized for leaf rolling at three distinct growth stages (tillering, booting, and spike emergence). The contact angle of leaf surface dynamics (adaxial and abaxial), water budget, and morphophysiological traits of genotypes were measured. The results indicate that leaf rolling varies from inward to twisting type among genotypes and positively affected the water use efficiency and soil moisture difference at all growth stages under normal and drought conditions. Results of wetting property (hydrophilic < 90°) of the leaf surface were positively associated with the atmospheric water collection (4–7 ml). The lower values of contact angle hysteresis (12–19°) also support this mechanism. Thus, genotypes with leaf rolling dynamics (inward rolled and twisted) and surface wettability is an efficient fog harvesting system in wheat for interception and utilization of fog water in drought-prone areas. These results can be exploited to develop self-irrigated and drought-tolerant crops.

**Supplementary Information:**

The online version contains supplementary material available at 10.1007/s11356-022-18936-2.

## Introduction

Climate change is one of the major concerns of today’s world due to anthropogenic activities that cause an increase in greenhouse gas emissions and extreme weather events like rising ambient temperatures, extreme precipitation events, and drought spells. These changes cause a 9–10% reduction in agricultural productivity and put food security at risk (Lesk et al. 2016; Rahman et al. 2018, 2020). Thus, the focus of modern research is to reduce the pressure on food security by using climate-smart strategies.

Among cereals, wheat is the most important due to its nutritional quality and potential to fulfill the energy demand of the ever-increasing world population. While it is predicted that the global population will reach 9.1 billion in 2050, there is a need to produce 70% more wheat to fulfill the increasing demands of exploding world population to ensure food security (FAO 2019). Ray et al. (2013) reported that global wheat production is 38% less than the projected demand (2050). The estimated global wheat production was 730.7 million tons in 2018–2019 (FAO 2019). Among many negative impacts of climate change, the unavailability of water is considered to be the most detrimental for the wheat crop (Asseng et al. 2015; Barnabás et al. 2008; Lesk et al. 2016; Rahman et al. 2021). Demirhan (2020) reported that a 1 °C increase in temperature will significantly reduce 4–6% global wheat production. Therefore, the development and adoption of climate-smart genotypes show better performance under climatic variations and enhance yield to ensure food security.

The most common responses of a wheat plant, when exposed to a water-stressed environment, include a reduction in the assimilation process, leaf expansion, stomatal conductance, and its association, which ultimately causes a decrease in yield (Bogale et al. 2011). Leaves are the main site of photosynthetic activity and provide the major source of photoassimilates for plant growth, development, and grain yield. Thus, the maintenance of leaves for photosynthesis has been advocated as a way of improving grain yield under drought stress. Leaf rolling is often described as a protective mechanism, i.e. a water conservation mechanism, which allows the maintenance of higher leaf turgor pressure during water stress and eventually delays the onset of senescence and increases the source-to-sink translocation of photoassimilates (Sirault et al. 2015). To counteract the adverse effects of drought stress, several morphological adaptations such as leaf rolling and leaf erectness are reported in cereals due to loss of turgor pressure and osmotic adjustment (Ali 2019; Blum 2011). Genetic diversity of leaf rolling in wheat (*Triticum aestivum* L.*)* has been reported (Sirault 2007), but studies investigating genotypic variation for leaf rolling in wheat plant leaves are rare. For the enhancement of yield, dynamic optimization of leaves structure is needed to maintain physiological integrity (Parry et al. 2011).

Adjustments for efficient photosynthetic activity in leaves are caused by the morphological diversity of leaf rolling (inward or outward) (Yuan et al. 2015). Leaf rolling slows down transpiration and enhances the accumulation of dry matter (Lang et al. 2004). Leaf rolling in the leaf blade is often due to lower water potential and turgidity of bulliform cells (Price et al. 1997; Xu et al. 2018). Moderate leaf rolling also ensures and maintains better water use efficiency compared to flattened and extremely rolled leaves (Juarez et al. 2004; Liu et al. 2016). Similarly, leaf rolling in wheat reduces the energy load on the leaf and lowers the surface temperature of the leaf while still allowing the light to go deeper into the canopy, which improves light interception and also reduces water loss (Rebetzke et al. 2001). Zhang et al. (2009) also revealed that moderate leaf rolling increases photosynthesis and yield.

The association of morphological adaptation and wetting mechanisms like fog drip and stem flow alleviates the drought effect and enhances leaf water relations, growth, and development processes of the plant (Eller et al. 2013; Gürsoy et al. 2017; Roth-Nebelsick et al. 2012). Some desert species such as cactus, grasses, and bushes intercept the atmospheric water by using hierarchical surface structures in combination with chemical properties for the transport of water. This moisture, once collected, can be stored and consumed. Studies related to rolling and its association with wettability shows that inward and coiled-shaped leaves help in the channeling and movement of water droplets such as inwardly rolled leaves of *S. crassa*, (Gürsoy et al. 2017), twisting-type leaves of *Stipagrostis sabulicola* (Roth-Nebelsick et al. 2012), and rolled leaf apex of xerophytic rosette families (Martorell and Ezcurra 2007). Therefore, leaf rolling is a significantly beneficial trait in wheat that can help to intercept atmospheric water toward the root zone in a similar manner to *Stipagrostis sabulicola* spp. in the Namib Desert (Roth-Nebelsick et al. 2012). Based on these observations, it is hypothesized that the leaf rolling dynamics of the wheat plant and their water retention ability can be helpful for self irrigation during fog events. Thus, the present study was designed to find out the atmospheric water harvesting by leaf rolling dynamics and also study their impact on physiological and yield-related traits under arid climatic conditions.

## Materials and method

### Site description and climate conditions

The study was conducted at the Institute of Plant Breeding and Biotechnology, MNS University of Agriculture, Multan (30° 10′ 53.25″ N, 71° 29′ 31.76″ E and 123 m elevation). The region has dry and semiarid agroclimatic conditions with abrupt changes in climatic patterns. Weather data (real time) of the last 11 years (2008 to 2018) shows that mean rainfall was 0.34 mm during the wheat growing season (November to April) (Fig. S1). Rainfall in November and December accounted for 0.02 and 0.32 mm, respectively, from March to April accounting for 0.62 to 0.35 mm. The average maximum and minimum temperatures were 26 °C and 13 °C, respectively, and the average solar radiation was 14.3 J/m^2^ during the wheat growing season. Hourly records of air and soil temperature (°C), rainfall (mm), relative humidity (%), leaf wetness (wet and dry minutes), wind speed (m s^−1^), wind direction (degree), and solar radiation (MJ m^−2^) were obtained from an automatic weather station (Campbell Scientific Inc., Logan, UT, USA) that was installed near the study sites (1–3 m).

### Plant material and study design

Fifteen wheat genotypes were selected from already characterized germplasm for novel leaf traits available in the Institute of Plant Breeding and Biotechnology, MNS University of Agriculture, Multan (unpublished data). For phenotyping, genotypes (Table S1) were sown in microplots (6 m^2^ of each genotype) under randomized complete block design (RCBD) with split plot arrangement (irrigation in main plot and genotypes in a subplot) in three replicates. There were two treatments, i.e., normal (4 irrigation including pre-sowing) and drought (1 irrigation at pre-sowing and 1 irrigation at tillering stage only). Row-to-row and plant-to-plant distances were maintained at 15 and 7 cm, respectively. Soil moisture contents and physiological parameters were recorded at three different stages (tillering (30 DAS), booting (75 DAS), and spike emergence (95 DAS)). Yield and yield contributing traits were also recorded. The same set of genotypes was also space planted (at 1 m^2^ plant-to-plant distance) in the field for atmospheric water harvesting by plant canopy.

### Characterization for leaf surface traits

Genotypes were characterized visually for the leaf rolling and leaf erectness. Leaf rolling was categorized based on a percentage of rolling of leaf margins. Inclination between the leaf blade midrib and the stem was done by dividing the vertical plane into four sectors of approximately 30°. All the microplots were scored by a single person to limit possible bias across the microplots. A scale was devised for this purpose explained as follows: leaf rolling was scored as 3 to 1 corresponding inward (> 67%), twisted type (34–66%), and outward leaf rolling (< 67%), respectively (Fig S2). Leaf angle was scored using a scale from 4 to 1 representing erect (30–40°), semi-erect (40–60°), semi droopy (60–90°), and droopy (60–90°). The scoring was repeated approximately for 3 consecutive days and resulted in a total of 45 scores of each trait.

### Measurement of soil moisture contents

For moisture contents, measurements were recorded using TDR-350 (Spectrum Technologies Inc., USA) from three different locations (start, mid, end) of a 5 m^2^ plot of each genotype as well as from the vicinity. The length of the probe was approximately 20 cm. TDR-350 measured the volumetric contents of the water in percentage, and its maximum water holding capacity was 22%. Root zone measurements were measured 2 cm away from the roots of the plant, while vicinity measurements were measured from a 30 cm distance from the plant. Average moisture contents for each genotype were estimated. Moisture difference was also calculated as follows:

$$\mathrm{Moisture difference }(\mathrm{MD}) =\mathrm{ moisture percentage of the root zone }(\mathrm{RZ}) -\mathrm{ moisture percentage of the vicinity }(\mathrm{V})$$

### Measurement of physiological parameters

The physiological parameters namely stomatal conductance (mmol H_2_O m^−2^ s^−1^), photosynthesis (μmol CO_2_ m^−2^ s^−1^), rate of transpiration (mmol H_2_O m^−2^ s^−1^), and water use efficiency (mmol CO_2_ mol^−1^ H_2_O) of the plants were recorded by using a CIRAS-3 Portable Photosynthesis System (Amesbury, USA). Measurements were taken between 10:00 am and 12:00 pm. The midportion of the top three leaves of the selected plant was kept in the leaf chamber during the measurements. Three observations were recorded from each genotype randomly, and the average was calculated.

### Contact angle measurement

Samples of the leaves were collected from the field for the measurement of contact angle. Static and dynamic contact angle measurements were performed by contact angle (Data Physics OCA 20, Filderstadt, Germany). For each sample surface, three measurements were taken at room temperature (18–20 °C) from the adaxial and abaxial surfaces of the leaf of each genotype. A total of 5–6 μl of demineralized water was applied to the leaf samples using an automatic dispense controller to measure static contact angle, and the measurement was taken immediately after the droplet was placed on the leaf sample. Dynamic contact angle measurements were measured to calculate the advancing (ACA) and receding contact angle (RCA) for determining the contact angle hysteresis (CAH). CAH of genotypes was done by increasing the volume of a demineralized water droplet from 2 to 5 μl and decreasing from 5 to 2 μl (Huhtamäki et al. 2018).

### Atmospheric water harvesting by plant canopy

Atmospheric water deposition and its movement at the leaves of wheat represent the atmospheric moisture harvesting performance (Hakeem et al. 2021). To determine water movement, collectors were constructed based on the requirements and procedures mentioned by Hakeem et al. (2021). Collector construction is shown in Fig. S3. During the measurement cycle, the known dry weight of cotton was placed at the base of each plant at 4:30 pm the day before sampling. The next day, early at 8:00 am, collectors were collected and weighed in the field immediately by using a portable weighing balance for the determination of the amount of absorbed water. A control collector was placed on a sheet adjacent to study plants (30 cm away from the plant canopy) to obtain control data for fog absorption by the collector material. The control collectors were also treated identically.

Water movement through the plant canopy (water that trickled down the leaves and was finally absorbed by the collectors during a measurement cycle) is presented in Fig. 2 and calculated as follows.

$$\mathrm{Water budget}=(\mathrm{wet weigh of collector }-\mathrm{ dry weigh of collector}) -(\mathrm{wet weigh of control collector }-\mathrm{ dry weigh of control collector})$$

To meet the objective of the study, available fog water was quantified and the association of water budget with soil moisture, leaf traits, and leaf wettability was determined. Genotypes were also phenotyped at tillering, booting, and spike emergence growth stages for leaf traits, moisture contents, and physiological parameters in drought conditions. Association of leaf rolling with morphophysiological traits in drought conditions was also determined.

### Statistical analysis

The heat map was developed for visualization of variation among genotypes by using a package heat map in R software (Barter and Yu 2018). The association of leaf surface traits with moisture contents and physiological parameters was drawn by using package cor and corplot in R software (Wei et al. 2017). Analysis of variance (ANOVA) was performed using package agricolea in R (de Mendiburu and de Mendiburu 2019). Mean data of different traits were used to make biplots for the screening of drought-responsive genotypes. Center and scale transformation were applied to data, and biplots were created by using the ggbiplot package of R software (Vu 2016). Data of contact angle was presented as mean ± SEM (standard error of the mean). Climograph (bar and line graph) of the means of climatic parameters on an hourly basis during the growing season (Nov.–Apr.) was developed in R by using packages plyr, dplyr, and ggplot2 (Tyner et al. 2017; Wickham and Francois 2014).

## Results

### Weather variables

The climograph presented in Fig. S4 indicates that fog occurred between 12 and 8 am during the night and early morning. Leaf wetness duration was maximum (11 h total) during December and January. Relative humidity was more than 80% during the wet hours and leaf wetness duration was 45 min during December, January, and February. Relative humidity and leaf wetness are the index fog harvesting and entrapment by leaf dynamics. Rainfall less than 0.25 mm was recorded. During these months, the minimum temperature fluctuated between about 10 and 15 °C from 12 to 9 am, while a maximum of 22–25 °C during the day (10 am to 4 pm). Soil temperature showed the same trend as air temperature.

### Atmospheric water harvesting and transportation

**Fig. 1:**
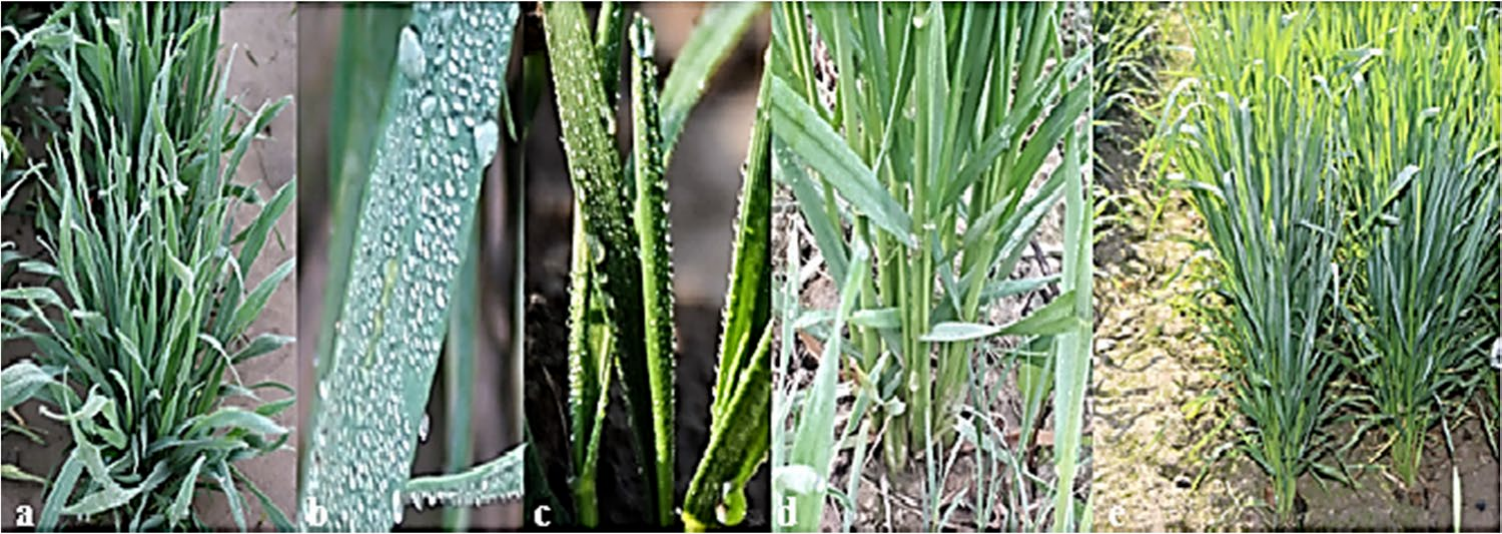
Wheat plant harvesting mechanism; **a** plant growing under fog events, **b** a leaf with fine droplets, **c** side view of a leaf showing attachment of droplets, **d** stem flow toward the base, and **e** moisture at the base of the plant

**Fig. 2:**
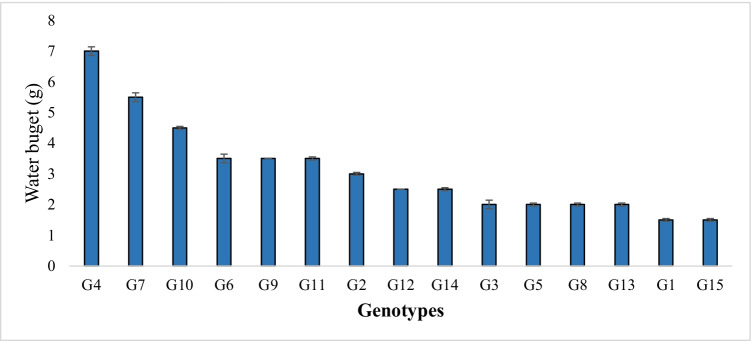
Water budget of selected 15 wheat genotypes in natural fog events and under field conditions. Results are presented as mean ± SEM

During fog events, it was observed that leaves of wheat plants collect a significant amount of water, which ran off through the leaf surface and stem toward the root zone resulting in the dampness of the soil (as presented in Fig. 1). The leaves of the plant canopy curl inward and outward from leaf margins and are shaped like twists. These curls enable them to channel toward the stem and then the root zone. Fog harvesting proceeds through the growth of tiny droplets attached to the edges and surface of the leaves (Fig. 1). These tiny droplets coalesce into large water drops rolling along the leaf surface and stem, which then dripped on the soil due to gravity. Leaf rolling resulted in a dual wetting property, which helps in droplet movements on the leaf surface. Erectness of leaves helps in the pinning of the droplets in the root zone of the plant, while bouncing/drip-off droplets occur from the tips of droopy leaves. This was further verified by the measurement of moisture difference and indicates that genotypes had 4–5% higher moisture within the root zone than vicinity. For the evaluation of moisture harvesting and its runoff on the wheat canopy, the water budget for genotypes was calculated and shown in Fig. 2. Weather variables during the attachment of collector till data recording time are presented in Fig. S5. The results indicate that genotype G4 had the highest water budget (7 g). The average water budget was 3.1 g. Genotypes G2, G6, G7, G9, G10, and G11 were above the average.

### Wettability of wheat leaf surface dynamics and its association with leaf rolling, physiological parameters, and water budget

**Fig. 3:**
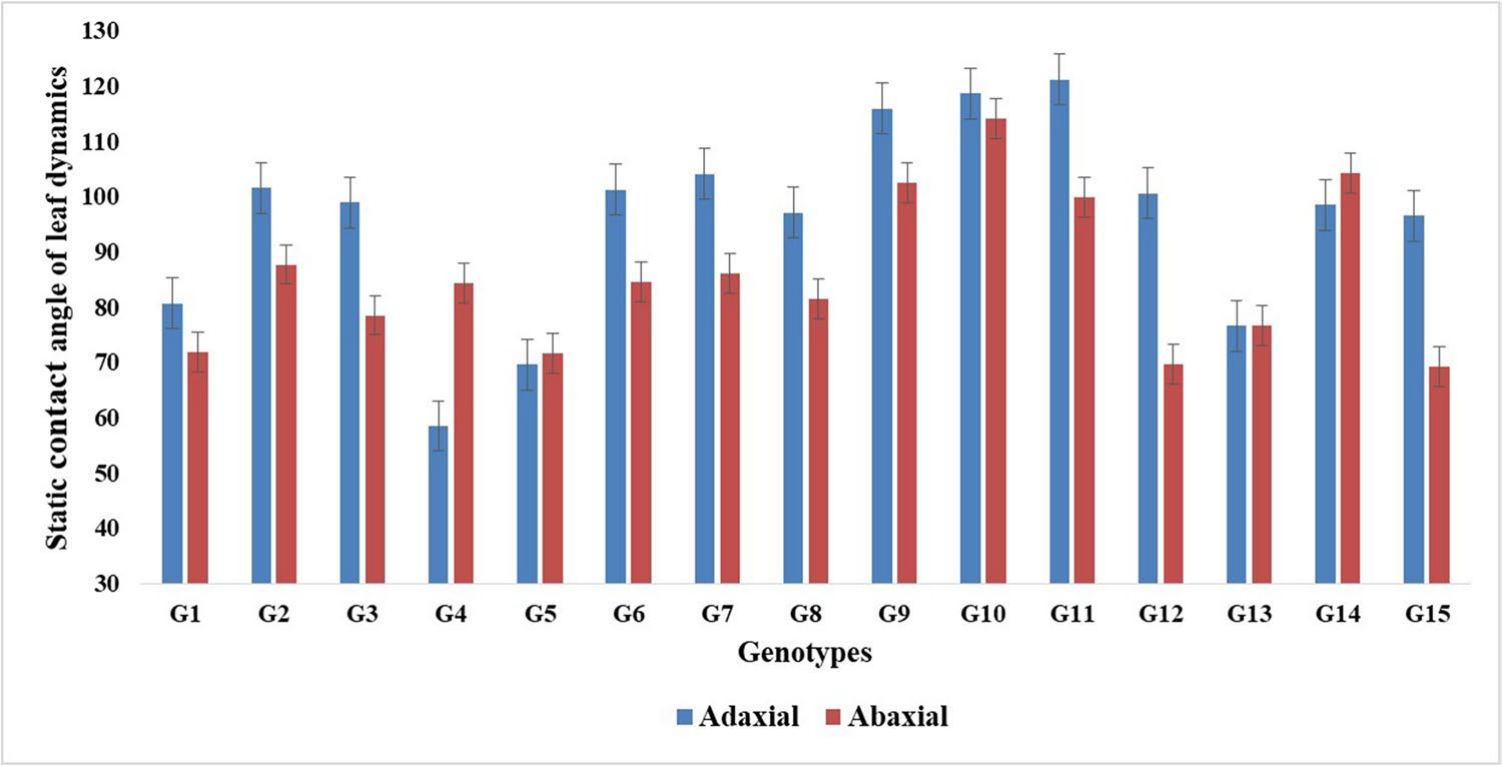
The static contact angle of leaf dynamics (adaxial and abaxial) of 15 wheat genotypes. Results are presented as mean ± SEM

The static contact angle of the adaxial and abaxial leaf surface of fifteen genotypes was measured (Fig. 3). A significant variation was observed among genotypes presented in Table S2. The mean static contact angle for both surfaces and their standard error are shown in Fig 3 and S6. The adaxial surface of four genotypes (G4 > G5 > G13 > G1) had wettability less than 90° and behaved as hydrophilic. Similarly, the mean contact angle of the abaxial surface shows that the abaxial surface of all the genotypes had wettability less than 90° except genotype coded as G9, G10, G11, and G14. Based on static contact angle information, three genotypes having hydrophilic properties for both surfaces were selected for dynamic contact angles. Advancing contact angle (θ_adv_), receding contact angle (θ_rec_), and contact angle hysteresis (CAH) of the abaxial and adaxial surfaces of the genotypes are given in Table 1. G1 had the lowest CAH values followed by G5 and G1, respectively.

**Table 1:** The dynamic contact angle of ab-axial and the ad-axial surface of wheat genotypes

Genotypes	Advancing contact angle (θ_adv_)		Receding contact angle (θ_rec_)		Contact angle hysteresis (CAH)	
	Abaxial	Adaxial	Abaxial	Adaxial	Abaxial	Adaxial
**G-1**	88 ± 0.42	115 ± 0.16	37 ± 0.16	78 ± 0.21	51± 0.05	37 ± 0.113
**G-4**	94 ± 0.14	60 ± 0.27	75 ± 0.14	48 ± 0.23	19 ± 0.08	12 ± 0.02
**G-5**	83 ± 0.14	113 ± 0.21	41 ± 0.21	85 ± 0.04	42 ± 0.06	28 ± 0.05

**Fig. 4:**
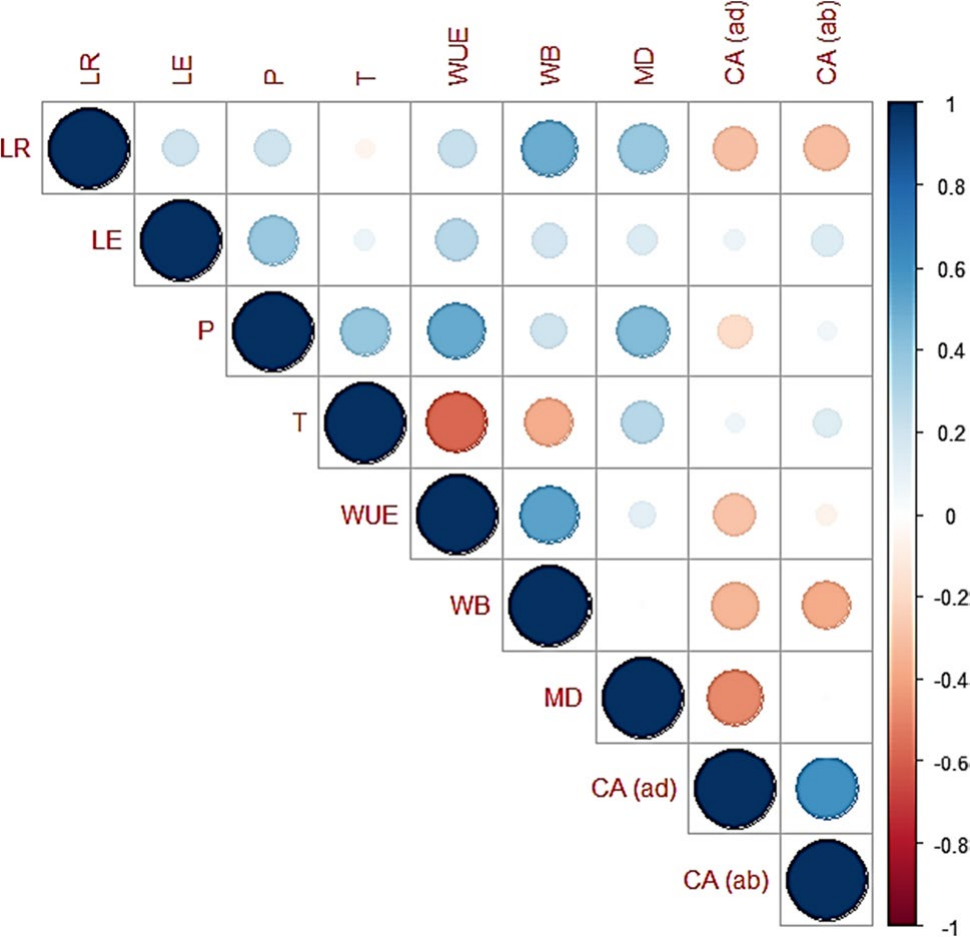
Correlation plot of leaf rolling (LR), leaf erectness (LE), water budget (WB), physiological parameters viz photosynthesis (P), transpiration (T) and water use efficiency (WUE), and contact angle (CA (adaxial) and (abaxial)) of 15 wheat genotypes. The blue shade shows a positive correlation, and the pink shade shows a negative correlation. The size of the circle shows how traits are associated with each other

A positive correlation was found between leaf rolling (LR) and leaf erectness (LR) (Fig. 4). The leaf traits were positively correlated with photosynthesis (P), water use efficiency (WUE), water budget (WB), and moisture difference (MD). LR was negatively correlated with transpiration (T) and contact angle (CA) of both surfaces, while LE showed a positive correlation with physiological parameters, WB, MD, and CA. Moisture difference and water budget were positively correlated with leaf traits, photosynthesis, and water use efficiency while negatively correlated with contact angle.

### Phenotyping under normal and drought conditions

**Fig. 5:**
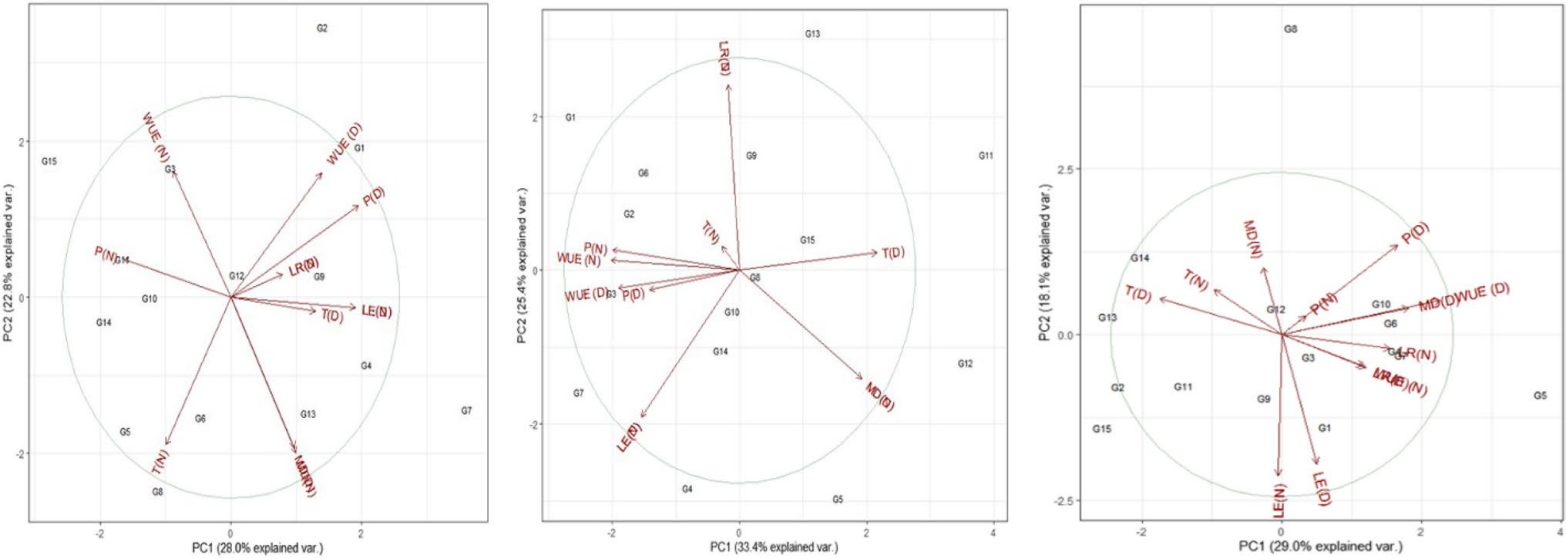
Biplot analysis of leaf traits viz leaf rolling (LR), leaf erectness (LE), physiological parameter viz photosynthesis (P), transpiration (T), and water use efficiency (WUE) of selected 15 wheat genotypes at tillering (**a**), booting (**b**), and spike emergence (**c**) stage grown under normal (N) and drought (D) field conditions. Circle explains the theoretical maximum extent of the arrows, added by the default confidence interval of 68%. Arrows show the correlation among the traits for their respective environment

**Fig. 6:**
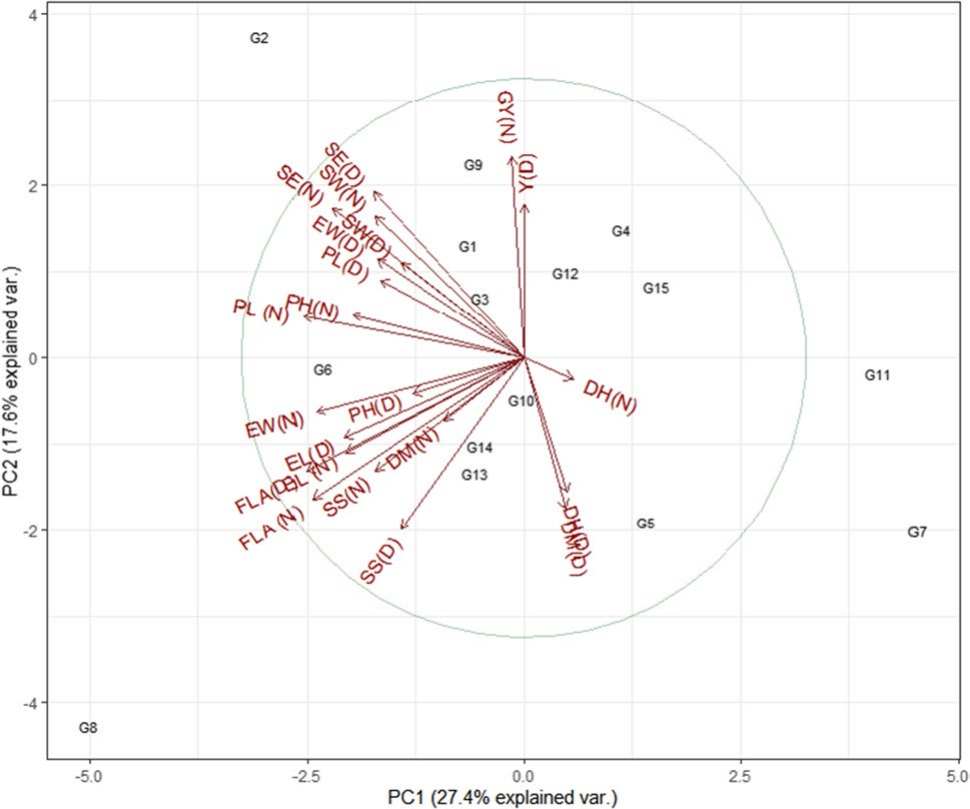
Biplot analysis of yield traits viz flag leaf area (FLA), peduncle length (PL), ear length (EL), plant height (PH), days to heading (DH), days to maturity (DM), ear weight/spike (EW), seed weight/spike (SW), spikelets/spike (S), no. of seed/ear (SE), and grain yield/plot (Y) of selected 15 wheat genotypes grown under normal (N) and drought (D) field conditions. Circle explains the theoretical maximum extent of the arrows added by the default confidence interval of 68%. Arrows show the correlation among the traits for their respective environment

A total of fifteen wheat genotypes were evaluated under normal and drought field conditions. Biplots of the fifteen wheat genotypes for all traits assessed at tillering, booting, and spike emergence are presented in Fig. 5, respectively. The average variability explained for all the traits was 52.2 at all growth stages. At tillering and spike emergence, vectors of LR, MD, and WUE had strong relations under drought (Fig. 5). Distribution and position of genotypes including G1, G4, and G7 at tillering and G5, G4, and G7 at spike emergences fall near to the vector of LR, MD, and WUE, indicating the response of these genotypes for leaf rolling and moisture harvesting. Comparatively, contrasting genotypes including G15 fall away from these vectors. At booting, vectors of LR, MD, and WUE had a weak relationship. The distribution of genotypes G1 and G7 fall near to the vectors of LR and WUE, indicating the response of genotypes for leaf rolling and water use efficiency. While contrasting genotypes including G5, G11, and G12 fall near to the moisture difference (MD) vector that indicated the response of these genotypes for moisture harvesting (Fig. 5). For yield and its components, the total variation explained by genotypes was 45% under both conditions (Fig. 6). The distribution and position of genotypes including genotype G2 and G15 fall near the vector of grain yield/plot (GY). For the others, vector seed weight (SW), no. of seed per ear (SE), and peduncle length (PL) genotypes including G7 and G8 indicated the response of these genotypes for yielding traits in wheat. Comparatively, contrasting genotypes include G7 and G11for GY, SW, SE, and PL.

### Phenotypic correlation

**Fig. 7:**
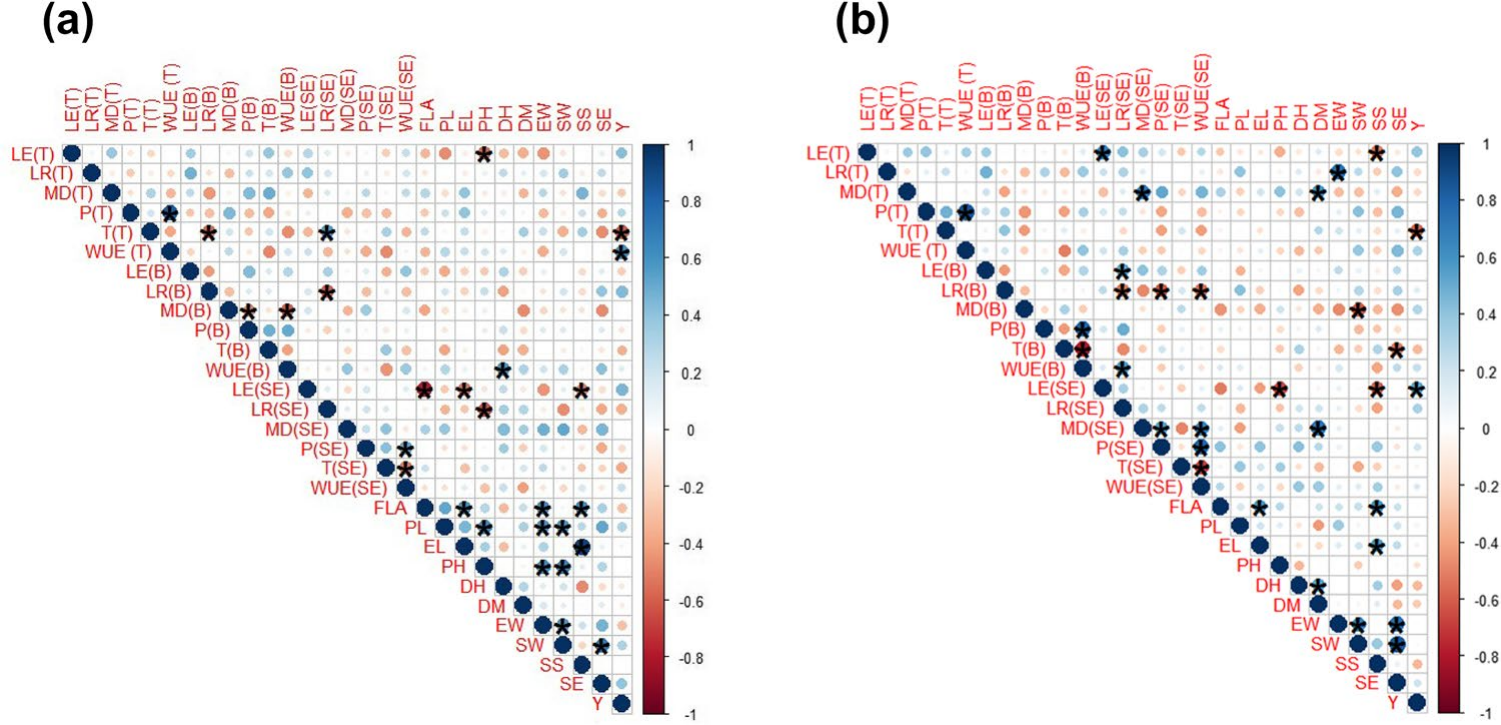
Correlation plot of leaf traits viz leaf rolling (LR), leaf erectness (LE), moisture difference (MD), physiological parameters viz photosynthesis (P), transpiration (T) and water use efficiency (WUE), and yield parameters viz flag leaf area (FLA), peduncle length (PL), ear length (EL), plant height (PH), days to heading (DH), days to maturity (DM), ear weight/spike (EW), seed weight/spike (SW), spikelets/spike (S), no. of seed/spike (SE), and yield/plot (Y) of 15 wheat genotypes grown under normal (a) and drought (b) field conditions. The blue shade shows a positive correlation, and the pink shade shows a negative correlation. The size of the circle shows how traits are associated with each other. *Indicates significant (*P* < 0.05) and without sign (*) indicates nonsignificant (*P* ≥ 0.05)

Correlation analysis between the leaf traits, moisture contents, physiological parameters, and yield parameters was carried out under the normal and drought field conditions for the evaluation of phenotypic association. Phenotypic correlations between all possible studied trait pairs at three growth stages are presented in Fig. 7. Under normal field conditions, leaf traits, LE at tillering, and LR at spike emergence were found to have a significant and negative correlation with PH. LR at booting was found negatively correlated with LR at spike emergence. Both traits at spike emergence showed a significant and negative correlation with yield traits. Moisture difference also showed a negative and significant correlation with P and WUE at the booting stage. Among the physiological parameters, WUE at tillering was found to have a significant and positive correlation with Y/P, respectively. At booting, WUE was also found to have a positive and significant correlation with DH. Similarly, at spike emergence, P and T were found to have a positive and negative but significant correlation with WUE. All the other studied traits were found to have nonsignificant correlations (Fig. 7a).

Under drought conditions, LE at tillering and booting was found to have a significant and positive correlation with LE and LR at spike emergence, respectively, and Y/P. While negative and significant was found with PH at spike emergence and SS at tillering and spike emergence. LR at tillering was also found significantly and positively correlated with EW. At booting, LR found a negative association with LR at spike emergence. Moisture difference at tillering and spike emergence was found to have a significant and positive correlation with MD and DM at tillering and P, WUE, and DM at spike emergence, while a negative correlation was found at the booting stage with SW. Physiological parameters showed a positive and significant correlation of P with WUE at all stages (tillering, booting, and spike emergence). While a significant and negative association was found for T with Y/P at tillering, T at booting and spike emergence also negatively correlated with WUE at both stages. Among yield traits, FLA was found to have a positive association with EL and SS, EL with SS, DH with DM, EW with SW and SE, and SW with SE. All other studied traits were found nonsignificant (Fig. 6b).

### Heat map of leaf traits, moisture difference, and physiological and yield parameters

**Fig. 8:**
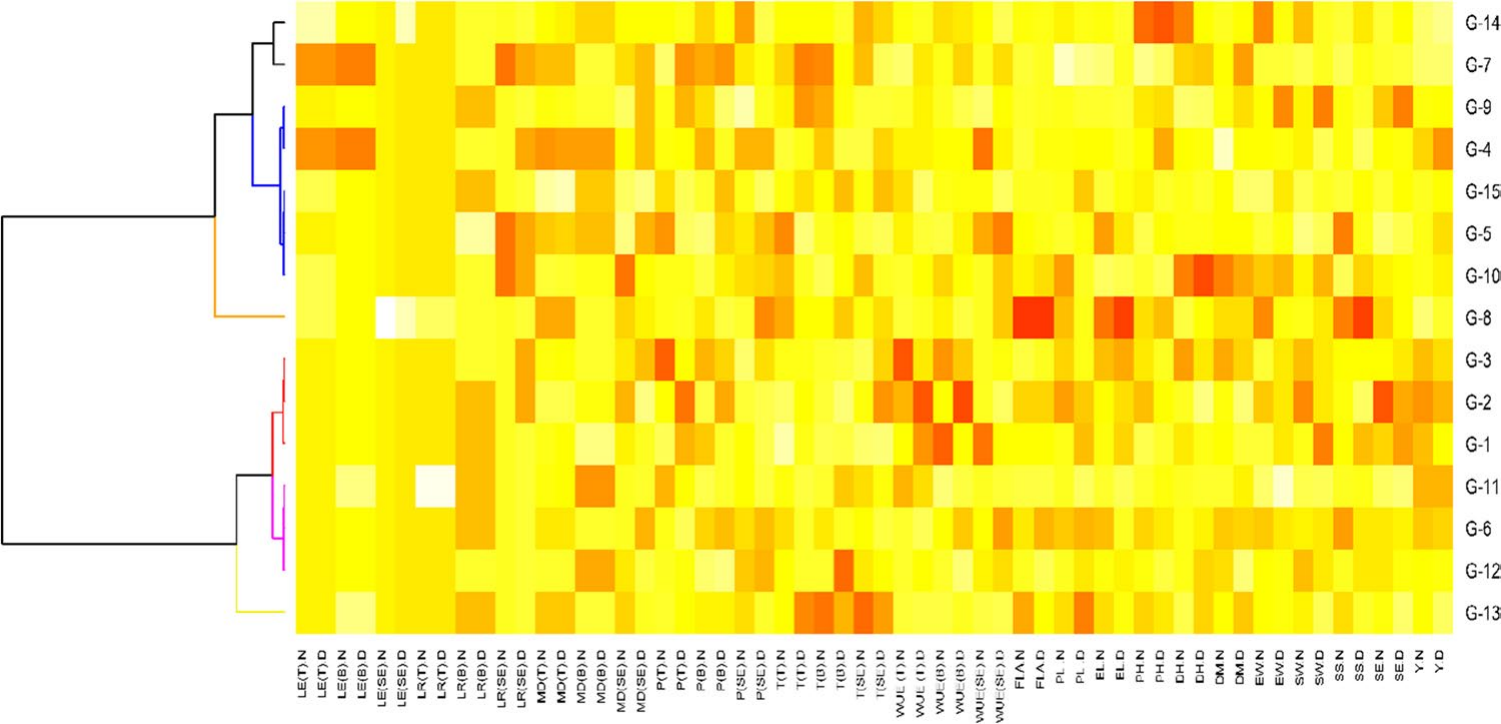
Heatmap of leaf traits viz leaf rolling (LR), leaf erectness (LE), moisture difference (MD), physiological parameters viz photosynthesis (P), transpiration (T) and water use efficiency (WUE), and yield parameters viz flag leaf area (FLA), peduncle length (PL), ear length (EL), plant height (PH), days to heading (DH), days to maturity (DM), ear weight/spike (EW), seed weight/spike (SW), spikelets/spike (SS), no. of seed/spike (SE), and yield/plot (Y) of 15 wheat genotypes grown under normal (N) and drought (D) field conditions

The heat map indicated the relative performance of the genotypes in normal and drought stress conditions for the studied traits (Fig. 8). It was noted that LE values showed a similar trend under normal and drought conditions for all genotypes; however, variations existed in growth stages. Similar to LE, the response of LR for all genotypes was similar at tillering and booting, while variation was shown at spike emergence. MD also showed variations among genotypes at all growth stages under both conditions, while among treatments, variations showed at spike emergence among genotypes. Physiological parameters, i.e., P, T, and WUE, showed higher values for most of the genotypes at all growth stages under drought conditions. Yield traits were depicted to have medium to higher values for most of the genotypes under drought stress.

## Discussion

Fog plays an ecological role as an alternative water source for plants (Burgess and Dawson 2004; Malik et al. 2014). The present study was focused on the atmospheric water harvesting and phenotyping of leaf rolling dynamics, and their association with morphophysiological parameters. As results indicated that wheat plants have leaf morphology for efficient moisture harvesting similar to other plants (Azad et al. 2015; Ju et al. 2012; Malik et al. 2014; Roth-Nebelsick et al. 2012; Sharma et al. 2018). The twisted (spiral) leaves of the wheat plant enable the water droplets to be channeled and pinned toward the root zone. The exploitation of the leaf rolling dynamics in wheat can increase the water status of plants to architect climate-smart wheat (Lopes et al. 2015).

Real-time data of climatic parameters recorded by the weather station revealed that atmospheric air becomes saturated with 80–100% water vapors and moisture in December, January, and February (Fig. 2). Similarly, it has been reported that in the Multan region, humidity increases up to 100% during foggy days (Yasmeen et al. 2012). Relative humidity (80–100) and leaf wetness (more than 11 h) is an index of the entrapment of water vapor on the leaf. However, climatic data of the Multan region shows that moderate to shallow fog persists, indicating that there is enough water in the atmosphere during the wheat-growing season when the crop is at the vegetative stage. During December, January, and February, leaf wetness was more than 45 min/day (Fig. S4). It revealed that when the wheat crop was at the vegetative stage especially at tillering, booting, and spike emergence stages, there was maximum atmospheric moisture in the form of fog and dew.

In this study, a set of 15 genotypes were evaluated under foggy events to quantify the amount of atmospheric water and its movement toward the base of the plant. Fog conditions are also described in Fig. S5. We found that the genotypes having inward and spiral/twisted-type leaf rolling and hydrophilic property had more quantity of water (4–7 g) at the base as compared to other genotypes (Figs. 2, 4, and 5). Similar results were found that desert grass *Stipagrostis sabulicola* spp. had erect/spiral-type leaves, and their association with other leaf traits help them in fog capturing and directional movement of water (Roth-Nebelsick et al. 2012). It shows that the wetted root zone of these genotypes was due to the atmospheric water input (Fig. 1). Positive association of water budget, moisture difference, and water use efficiency with leaf rolling revealed that atmospheric water harvesting was enhanced with the leaf rolling (twisting type to inward rolled) (Fig. 4). These results were further verified by the evaluation of these genotypes with moisture contents under drought. The association of the traits of these genotypes with physiological parameters at tillering, booting, and spike emergence stage shows that these genotypes also have more net photosynthesis rate (5.5, 8.4, 7.5 μmol CO_2_ m^−2^ s^−1^, respectively), water use efficiency (5.3, 5.9, 3.6 mmol CO_2_ mol^−1^ H_2_O, respectively), and low transpiration rate (0.9, 1.4, 1.3 mmol H_2_O m^−2^ s^−1^, respectively). Liang et al. (2002) revealed that the decreased water consumption mainly resulted from the decreased transpiration rate in wheat. During the recovery of soil moisture, the transpiration rate through stomata could return to normal during wheat growth. So, this set of genotypes can be used as a guide to water-saving plants.

The mechanism of fog harvesting in plants consisted of water capture on the leaf surface, coalescence, and removal from the surface. The wettability of a surface can be classified as either hydrophilic, hydrophobic, or super-hydrophobic when the contact angle of water is < 90°, > 90°, and > 150°, respectively (Gürsoy et al. 2017; Huhtamäki et al. 2018). The purpose of the additional characterization of the leaf by CA was to confirm the wettability of the leaf surface and the acquisition of fog water within the root zone of the plant. In our results, it was found that both hydrophobic and hydrophilic surfaces existed in wheat (Fig. 3). The negative association of contact angle of both surfaces with leaf rolling and water budget also revealed that leaf rolling (twisting type to inwardly rolled type) along with hydrophilic property in wheat enhanced the atmospheric water harvesting (Fig. 4). The efficiency of both surfaces was compared with the water budget; it reveals that hydrophilic property improved the collection efficiency in wheat (Fig. 3 and 4). Results also showed that hydrophilic surfaces had a higher affinity than hydrophobic surfaces for the impinging of atmospheric water droplets and droplets rolling. These results show that the wetting mechanism in wheat takes place through leaf water repellency and stem flows that eventually increased the soil moisture within the root zone (Rosado and Holder 2013). So, the wheat canopy can be modified with twisted and inward-type rolling and hydrophilic properties to improve fog collection efficiency.

The development of leaf traits and soil moisture variation ultimately affect the growth and yield. But many times, their reliability for effective phenotyping remains doubtful. This is because the outcomes are highly correlated with the stage of plant growth and development (Flohr et al. 2018). It means that leaf traits showing the harvesting of atmospheric water at tillering stage may not harvest water at the booting or spike emergence stage. The characterization of leaf traits showed that inward and erect canopy at tillering and spike emergence (G7 and G5) captured more water (10% and 14%, respectively), while twisting-type and erect canopy (G12) captured maximum moisture (4.3%) at booting. Similar results were also explained by Sharma and Dunn (1969); they revealed that plants changed their architecture with changes in their growth stages and environmental conditions.

Variations among leaf traits, moisture difference, physiological parameters, and yield-contributing traits indicated by a heat map (Fig. 8) showed that genetic diversity exists for the collection and channeling of atmospheric moisture among genotypes. Crop productivity and yield can be limited by insufficient net photosynthesis in response to water stress. Leaves are the main site of photosynthetic machinery that ultimately affects the growth, development, phenology, and yield of cereals (Pandey and Shukla 2015; Ul Hassan et al. 2021). In this study, physiological parameters at different growth stages were recorded, and it was observed that they had a differential response as it was changing with genotype and also with the growth stage (Figs. 5 and 8). It indicated that their response was dependent largely on the growing conditions and stress.

The efficiency of breeding programs in diverse environments can be improved by gaining an understanding of the associations between grain yield and different morphophysiological traits. In this study, several morphophysiological traits were used for the correlation with yield under drought stress conditions (Fig. 7). Positive correlations were found between yield (ear weight, no. of seed/ear, and yield/plot) and other physiological parameters (water use efficiency) as well as the number of spikes per plant under drought. It revealed that drought stress increased the ear weight, no. of seed/ear, and yield in water-saving genotypes at tillering stage. Similarly, correlation showed that yield was also positively correlated with water use efficiency at both booting and spike emergence stages (Fig. 7). These results suggested that water use efficiency may be an important factor influencing photosynthesis in wheat. Furthermore, negative correlations were found between yield and plant height, peduncle length, and days to maturity. Altogether, this suggests that this set of genotypes will be used in the future breeding program to develop high-yielding wheat genotypes with the increased number of spikes, water use efficiency, as well as decreased plant stature.

Under drought conditions, results of the association of moisture contents and physiological parameters with leaf rolling dynamics reveal that the expression of leaf rolling was dependent on the availability of moisture contents. Less water within the root zone increased the rolling index of the leaf edges (Araus 2008). The negative association of leaf rolling with physiological parameters shows that if leaf rolling increases from flattened to rolled type, it causes the reduction of photosynthesis, transpiration, and ultimately water use efficiency due to the less exposure of the leaf surface. It indicated that their response was dependent largely on the growing conditions and stress-enhanced rolling index in the leaves. Similar results were also found by Rebetzke et al. (2001) that leaf rolling expression depends on the evaporative demand and heterogeneity of soil water content. So, results indicate that inward and twisted leaf rolling are required for fog capturing under restricted irrigation.

Under restricted irrigation, genotypes were also evaluated for yield-related traits, and the result shows that efficient moisture-harvesting genotypes (G1, G4, and G12) performed superior for yield under drought (Fig. 5). These water-saving genotypes also performed superior for more moisture difference, photosynthesis, water use efficiency, and transpiration rate (Fig. 4). These results support that spiral-type rolling enhanced the water harvesting and its acquisition within the root zone and increased the plant water status that ultimately causes the improvement of the overall plant growth and results in yield improvement. The importance of these traits and their association with atmospheric water alleviates the drought effect and enhances leaf water relation, growth, and development is quite clear in our results as well as in previous studies (Eller et al. 2013; Roth-Nebelsick et al. 2012).

## Conclusion

Water availability for wheat crops in wheat-growing regions is predicted to decrease as the intensity of water stress increases. Atmospheric water harvesting is an economical and substantial source of water in arid and semiarid regions. Under this scenario, plant adaptations are needed for the atmospheric water acquisition in wheat. Leaf traits like leaf rolling dynamics, leaf angle, and leaf wettability determine the acquisition of fog water in wheat. Our findings indicate that inward to twisted leaf canopy can help capture and retain atmospheric water within the root zone. It is a function of water retention due to stem flow and droplet movement resulting from lower contact angle hysteresis. Leaf rolling dynamics (inward rolled and twisted) and surface wettability are efficient atmospheric water harvesting systems in wheat for interception and utilization of fog water in drought-prone areas. Diversity for the leaf rolling dynamics also exists that allows breeding for climate-smart wheat. It is suggested that wheat germplasm may be screened to find out the self-irrigated wheat genotypes, which capture fog water through a stem flow. These genotypes with an efficient fog-capturing canopy would be hybridized, so that new self-irrigated phenotypes may be generated. Self-irrigated wheat genotypes would be better adapted in arid and semiarid regions. This approach can produce a good yield with better water use efficiency.

## Supplementary Information

Below is the link to the electronic supplementary material.

Supplementary file1 (DOCX 703 KB)

## Data Availability

The datasets and codes used and/or analyzed during the current study are available from the corresponding author on reasonable request.
