# Supplementary material for: Leaf rolling dynamics for atmospheric moisture harvesting in wheat plant as an adaptation to arid environments
Source: Environ Sci Pollut Res Int. 2022 Feb 25;29(32):48995–9006. doi: 10.1007/s11356-022-18936-2 (PMC9252964; doi:10.1007/s11356-022-18936-2)
Supplement: Supplementary file 1 — Supplementary file1 (DOCX 703 KB) [file 11356_2022_18936_MOESM1_ESM.docx]

Leaf rolling dynamics for atmospheric moisture harvesting in wheat plant as an adaptation to semi arid environments

**Supplementary Material**

**Table S1: List of wheat genotypes**

| **Code** | **Genotype** | **Pedigree** |
| --- | --- | --- |
| G-1 | B-3 | CROW'S'/NAC//BOW'S'PB 22138-3A-0A-0A-234A-0A |
| G-2 | B-6 | SU-7-63-0951 |
| G-3 | B-8 | 29SAWSN11-12/54 |
| G-4 | B-9 | SU-7-56-0806 |
| G-5 | 8 | SU-7-60-0881 |
| G-6 | A-32 | PRL/PASTOR//2236(V6550/SUTLEH-86) |
| G-7 | A-85 | AARI-1/11-12 |
| G-8 | A-131 | 27 HTN/1-54 |
| G-9 | F2-109 | (1915×ESWYT-4)×1903 |
| G-10 | F2-149 | (1305×NAX-1)×9478 |
| G-11 | ZA-7 | 12-1058 |
| G-12 | 241 | 12-1015 |
| G-13 | 366 | 12-1148 |
| G-14 | Chakwal-86 | FORLANI/ACC//ANA 75SWM4578-56M-3Y-3M-0Y-0PAK |
| G-15 | Ujala-16 | KIRITATI/4/2*WEAVER/TSC//WEAVER/3/WEAVER |

**Table S2:** Mean squares of the leaf surface of 15 wheat genotypes

| **Source of Variation** | **df** | **Abaxial leaf surface** | **Adaxial leaf Surface** |
| --- | --- | --- | --- |
| **Replication** | 1 | 0.56 | 1.08 |
| **Genotypes** | 14 | 581.12*** | 944.98*** |
| **Residuals** | 29 | 1.36 | 0.57 |

*** indicates significant differences at p≤ 0.001 and without sign (*) indicates non-significant.

**Table S3:** Mean squares of various yield-related traits of 15 wheat genotypes grown in normal and drought stress.

| **Source of Variation** | **df** | **FLA** | **PL** | **EL** | **PH** | **DH** | | **DM** | **EW** | **SW** | **SS** | **SE** | **GY** |
| --- | --- | --- | --- | --- | --- | --- | --- | --- | --- | --- | --- | --- | --- |
| **Block** | 1 | 38.66 | 0.11 | 0.49 | 112.61 | 534.02 | | 263.20 | 1.35 | 0.31 | 2.67 | 799.35 | 8641215 |
| **Irrigation** | 1 | 315.01 | 1.80 | 1.12 | 9.76 | 8.82 | | 56.71 | 1.35 | 0.26 | 0.19 | 212.82 | 15682 |
| **Residual (a)** | 1 | 2.58 | 7.92 | 0.07 | 9.44 | | 22.82 | 4.45 | 0.42 | 0.07 | 2.67 | 79.35 | 12042 |
| **Genotypes** | 14 | 1061.79*** | 40.79*** | 8.47*** | 384.44*** | | 20.24** | 28.49* | 1.34* | 1.26* | 7.63* | 492.51** | 1568009*** |
| **Irrigation x Genotypes** | 14 | 15.77 | 7.85 | 2.70* | 15.76 | | 8.21 | 4.28 | 0.39 | 0.70 | 2.32 | 105.39 | 46034 |
| **Residual (b)** | 28 | 47.32 | 9.73 | 1.04 | 41.50 | | 5.38 | 10.76 | 0.56 | 0.53 | 3.62 | 129.39 | 138460 |

*, **, *** indicates significant differences at p≤ 0.05, p≤ 0.01, p≤ 0.001 and without sign (*) indicates non-significant. Yield contributing traits flag leaf area (FLA), peduncle length (PL), ear length (EL), plant height (PH), days to heading (DH), days to maturity (DM), ear weight/spike (EW), seed weight/spike (SW), spikelets/spike (S), no. of seed/spike (SE) and yield/plot (Y) of selected 15 wheat genotypes grown under normal (N) and drought (D) field conditions.


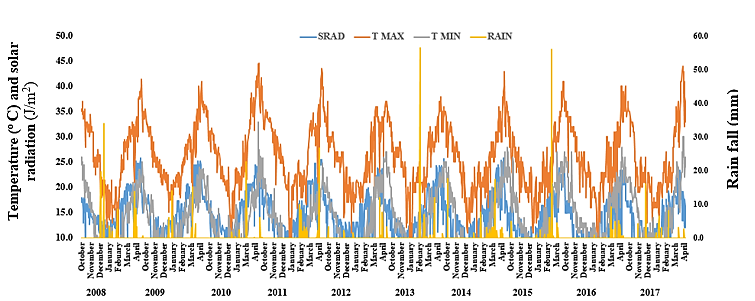


**Figure S1.** The trend line of climaric parameters (from year 2008 to 2017) viz solar radiation (SRAD), maximium temperature (T MAX), minimium temperature (T MIN), rainfall (RAIN) during wheat growing season


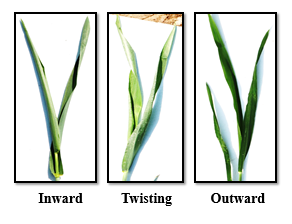


**Figure S2**. Leaf rolling dynamics of wheat leaf surface


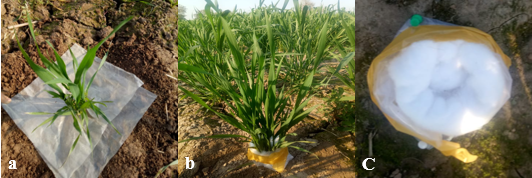


**Figure S3.** **Collector construction for fog capturing.** (a) The base of the plant is covered with a plastic sheet. (b) The cotton collector was clamped around the stem of the leaves at the base of the plant and covered from all sides by plastic tape. (c) Control collector.


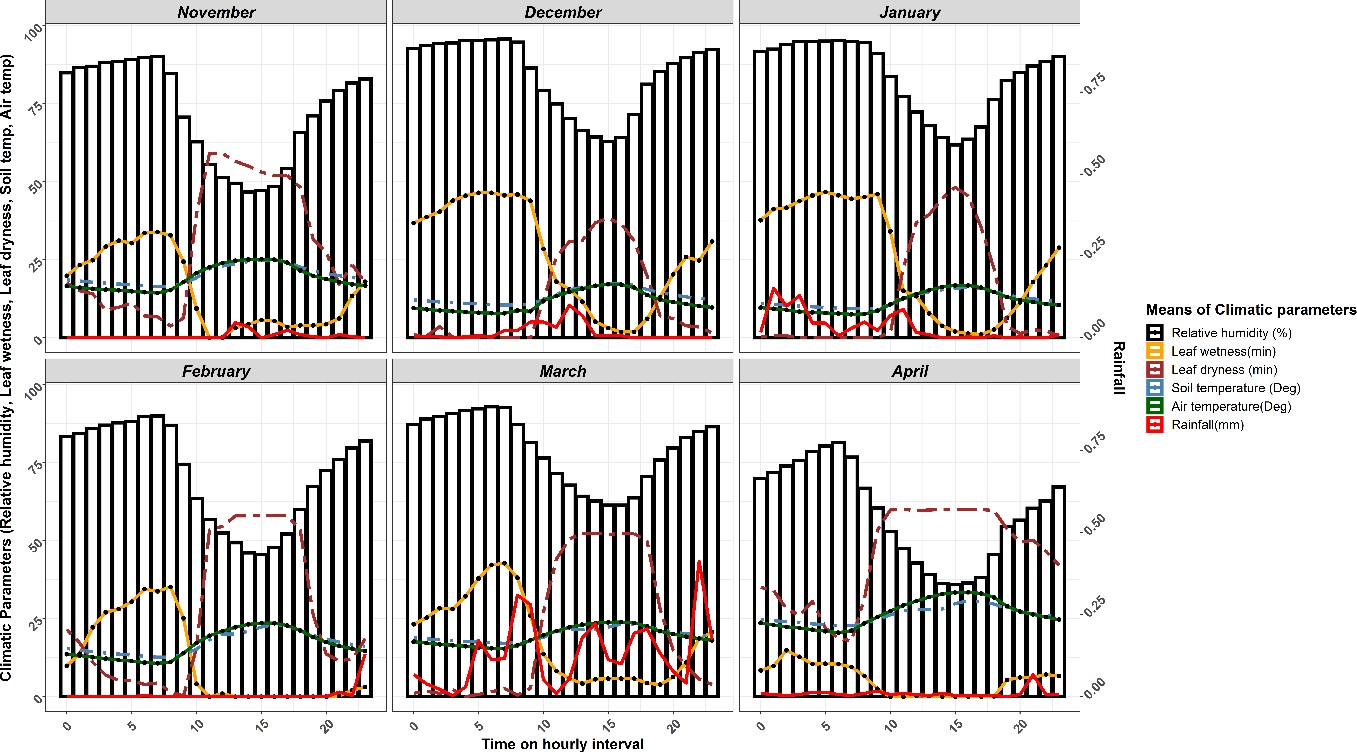


**Figure S4.** Daily average weather variables for the wheat-growing season (November-April) during 2019-20

**Figure S5.** The trend line of real-time data of climatic parameters viz leaf dryness (LD), leaf wetness (LW), relative humidity (RH), wind speed (WS), solar radiation (SR), air temperature (AT), rainfall (R), soil temperature (ST) from the time of collector placement to sampling on 16^th^ and 17^th^, January 2020.


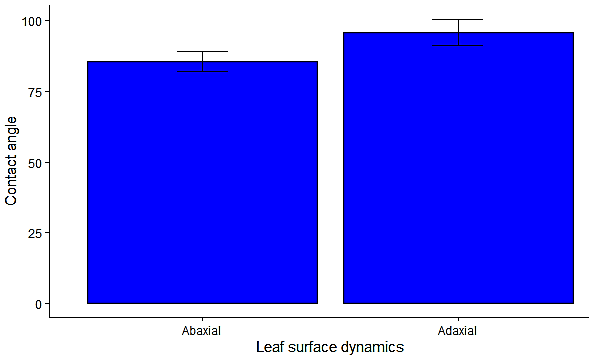


**Figure S6.** The static contact angle of wheat leaf surface (abaxial and adaxial). Results are presented as the mean of both surfaces ±SEM (standard error of the mean).
